# Fortified Nudges? Protecting the Vulnerable in a Post-COVID Society

**DOI:** 10.1007/978-3-030-65355-2_24

**Published:** 2021-03-20

**Authors:** Robin Pierce

**Affiliations:** 1 Tilburg University, Tilburg, The Netherlands; 2 Tilburg University, Tilburg, The Netherlands; 3 Tilburg University, Tilburg, The Netherlands; 4 Tilburg University, Tilburg, The Netherlands; Tilburg Institute of Law, Technology and Society, Tilburg Law School, Tilburg, The Netherlands

## Abstract

The eagerly awaited transition back into a functioning and vibrant society presents numerous challenges, not the least of which is how to protect the vulnerable. As society emerges from the “crisis” phase and the lockdown is lifted, it remains unclear to what extent it should be left to the vulnerable to protect themselves.

The elderly, the infirm, and those with existing health conditions are particularly susceptible to tragic outcomes from the coronavirus. To be vulnerable to a severe impact of COVID-19 turns the disease into a pervasively lurking death threat. Yet, to suggest that the vulnerable spend their lives in retreat in order to significantly minimize the risk is problematic in multiple ways. As policy-makers craft the way forward, the question must be asked whether an appeal to the goodwill and voluntary cooperation of people, along with the slow evolution of social norms, is an adequate approach to protecting the vulnerable.

The eagerly awaited transition back into a functioning and vibrant society presents numerous challenges, not the least of which is how to protect the vulnerable. As society emerges from the “crisis” phase and the lockdown is lifted, it remains unclear to what extent it should be left to the vulnerable to protect themselves.

The elderly, the infirm, and those with existing health conditions are particularly susceptible to tragic outcomes from the coronavirus. To be vulnerable to a severe impact of COVID-19 turns the disease into a pervasively lurking death threat. Yet, to suggest that the vulnerable spend their lives in retreat in order to significantly minimize the risk is problematic in multiple ways. As policy-makers craft the way forward, the question must be asked whether an appeal to the goodwill and voluntary cooperation of people, along with the slow evolution of social norms, is an adequate approach to protecting the vulnerable. Already, some lockdown measures are showing signs of durability, likely to last beyond the crisis needs. For example, avoidance of rush hours, tech-facilitated remote meetings, and working from home have all demonstrated a degree of success sufficient to question whether the “old common” continues to be necessary or is even preferable. For not only can these measures be cost-saving, climate-friendly, and more efficient, they also act against the spread of the virus.

But even as the “new common” way of doing things is embraced, some of the “old common” is likely to return, both as a matter of practicality and of preference. Leaving our homes to enjoy the theater, a dinner on the town, or a trip to the library are likely to have few satisfying substitutes. But what about the vulnerable among us? What is the nature of societal responsibility to protect the vulnerable in the post-COVID society, and what kinds of approaches will lead to an optimal balance of rights, liberties, and interests that do not place the vulnerable at undue risk of a life-threatening disease? This short essay offers Fineman’s notion of “inevitable dependency” as a basis for society’s obligation to create protective measures that do more to ensure the safety of the vulnerable compared to those that rely heavily on the goodwill of individuals. Measures that balance the two fundamental interests of protection of life and protection of freedom should lead us in creative directions that support goodwill with meaningful action. This essay proposes “the fortified nudge” as a step in this direction.

## Inevitable Dependency

Political philosopher Martha Fineman offers the concept of inevitable dependency as a feature of what it is to be human in society. Essentially, the term refers to the fact that all members of society will experience a period of dependency that will make them vulnerable in ways that the rest of society is not (Fineman 2017). This dependency, she argues, is inevitable. Though very few of us may navigate through life with minimal dependence and vulnerability, as human beings, we will all experience it.

In the post-COVID society, there will be the familiar vulnerabilities of frailty, disease, social and economic disadvantage, and so on, but there will be new forms of vulnerability occasioned by COVID-19 and its processes—how it spreads, the long-term effects, and how it impacts those afflicted with it. In many cases, the existing or pre-COVID vulnerabilities provide fertile ground for severe disease impact. However, COVID-19 creates new categories of vulnerability by its impact on the body. These can include damage to heart tissue (Wang et al. 2020) or lungs (Wilson 2020; Spagnolo et al. 2020) or exploiting weaknesses brought about by diabetes, cardiovascular disease, and other co-morbidities. In America, COVID-19 has exacted a particularly heavy toll on African-Americans (Price-Haywood et al. 2020). Some countries have noted a particularly harsh impact on persons with dementia (Yao et al. 2020), including those living at home. For persons having one or more of these vulnerabilities, the consequences of contracting the coronavirus could be fatal. How we protect the COVID-vulnerable in a post-COVID society will be a critical feature of a successful transition.

There are many challenges in knowing when and how to re-open, not the least of which is what will constitute sufficiently protective measures such that it will not unnecessarily cost lives. This is a matter of policy. What will it take to support our return to living as a connected and vibrant larger social community and what should be the responsibility of the state and the larger society? While voluntary acts are welcome, expecting the COVID-vulnerable to rely on the goodwill of others when their lives may be at stake seems unreasonable. Something more reliable and predictable is necessary.

## Insufficiency of Voluntary Measures

While nudges can be justified in a post-COVID society, such essentially voluntary measures are unlikely to be sufficient to minimize the risk to the COVID-vulnerable when the majority of people in that society no longer perceive a serious threat. The incentive to maintain social distancing behaviors is likely to diminish when such awkward behaviors no longer serve immediate interests. Nevertheless, while the most serious threat has passed for most people in society, the vulnerable remain at the mercy of this lurking decimator. Some who fall into this category have become resigned to a life in retreat, some for the remainder of their lives. Society owes more to these individuals than an offer “to pick up something at the store or pharmacy.” A life in isolation due to heightened risk that disproportionately falls to the lot of the vulnerable is unfair. In a post-COVID society, inevitable dependency, here perhaps best articulated as inevitable vulnerability, requires more than mere encouragement to behave responsibly to protect those at heightened risk. Rather, inevitable vulnerability would suggest a new form of self-interest that expands immediate self-interest, is more personal than “enlightened self-interest” (in which one sees the benefit to oneself in benefitting others) and casts the actors as responsible stewards investing in their own futures as well as those of the vulnerable. Moreover, the failure to do so would result in a profoundly marginalized status for the COVID-vulnerable as their ability to participate in the life of society would be substantially diminished. Most Western societies have crossed this terrain with mandatory accommodations for persons with disabilities. However, unlike persons with disabilities, the COVID-vulnerable are merely at risk, not disadvantaged in any manifest sense. They can go shopping, visit the library, and get to and from work with no special assistance. What differs for them from the majority in a post-COVID society is the level of risk and the likely consequences should that risk materialize. Thus, the harm is actually speculative, although grave. It is the gravity of this harm that justifies measures beyond voluntary action.

## Fortified Nudges

A fortified nudge would appear as one of many variations of the nudge, a concept introduced by Thaler and Sunstein in 2005 (Thaler and Sunstein 2009). It refers to state action that uses insights from behavioral economics to influence behavior such that people act in their own self-interest but also preserves choice. A classic example is placing fruit and vegetables in front of the pizza in the cafeteria. The choice is preserved, but patrons have been nudged to choose the healthier option. This strategy could be used to influence people to behave in ways that are less likely to spread the virus. We see this in the use of sidewalk distance bubbles, design artifacts indicating where people should stand to minimize contagion. A fortified nudge would take this design artifact a step further such that standing closer than the recommended 1.5 m is quite difficult. This is moving toward Lessig’s regulatory tool of “code” or “architecture,” in which the environment is designed in such a way that choice is largely or completely eliminated (Lessig 2003). There are many challenges to architecture, including legitimacy because often the compulsory behavior is being mandated by private parties and the required action is in their interests. By contrast, a fortified nudge would maintain the essential qualities of a nudge, performed by the state in the best interests of the people whose behavior is being influenced, but the fortified nudge, while preserving choice, takes a stronger hand in the steering of behavior. Thus, social distancing measures authorized by the state would also make another choice more difficult in some way. If proven effective, these measures might include the physical reduction of capacity on public transportation or browsing alternatives in the library that do not require touching. Thus making a trip to the library an enjoyable activity for the COVID-vulnerable, who would not have to risk their lives to do so.

The point is that if the COVID-vulnerable is to have a chance to flourish and participate in the life of society, measures need to be sufficiently robust to consistently and reliably reduce the risk to this segment of the population. Goodwill and voluntary efforts can go a long way, but the consistency of such measures is essential to transitioning to a fair, inclusive, and well-functioning society.
